# Salmonella Meningitis Complicated by Ventriculitis and Brain Abscesses in an HIV Positive Adult Patient

**DOI:** 10.7759/cureus.11223

**Published:** 2020-10-28

**Authors:** Tamoor Ahmed, Taha Ahmed

**Affiliations:** 1 Internal Medicine, King Edward Medical University/Mayo Hospital, Lahore, PAK; 2 Internal Medicine, University of Kentucky College of Medicine, Lexington, USA

**Keywords:** salmonella, bacteremia, hiv, ventriculitis, brain abscess, aids, antiretroviral therapy

## Abstract

Salmonella meningitis is a rare complication of Salmonella sepsis and is mostly reported in infants and young children. The incidence of Salmonella bacteremia is increased in immunocompromised adult individuals, such as those having human immunodeficiency virus (HIV) infection. Ventriculitis and brain abscess as a complication of Salmonella intracranial infection is particularly rare, even in patients who are immunosuppressed. Herein, we report a case of Salmonella meningitis complicated by ventriculitis and two brain abscesses in an HIV positive adult patient requiring mini-craniectomy and drainage along with a prolonged course of antibiotic therapy with a favorable outcome.

## Introduction

Salmonella infections can be enteric or non-enteric (abscesses, mycotic aneurysms, pneumonia, osteomyelitis, septic arthritis, endocarditis, and meningitis) or systemic (bacteremia) [1]. As with all opportunistic infections, HIV-positive patients are at increased risk of developing Salmonella bacteremia [2]. Intracranial infections are unusual manifestations of Salmonella bacteremia, and brain abscess is a very rare complication. The first case of Salmonella meningitis was reported by Gohn in 1907, whereas brain abscess due to Salmonella was first reported by Burrows in 1959 [3,4]. We describe a case of Salmonella bacteremia with meningitis complicated by ventriculitis and brain abscess in an immunocompromised male. 

## Case presentation

A 44-year-old man with a history of HIV infection/AIDS diagnosed more than 10 years ago, unknown brain tumor post-resection 19 years ago, presented to the emergency department with acute onset confusion and right-sided hemiparesis. As per his mother, he had slurred speech a few hours prior to the presentation, and she denied any previous gastrointestinal or cardiorespiratory symptoms. He had not been compliant with his HIV antiretroviral therapy (ART) and had stopped them a few months back. On presentation, the patient was afebrile with a temperature of 99.9°F, blood pressure 142/92 mmHg, heart rate 85 beats per minute, and respiratory rate of 20 breaths per minute. Physical examination revealed expressive aphasia and hyperreflexia with clonus in the bilateral lower extremities. A fundoscopic exam ruled out HIV retinopathy.

**Figure 1:**
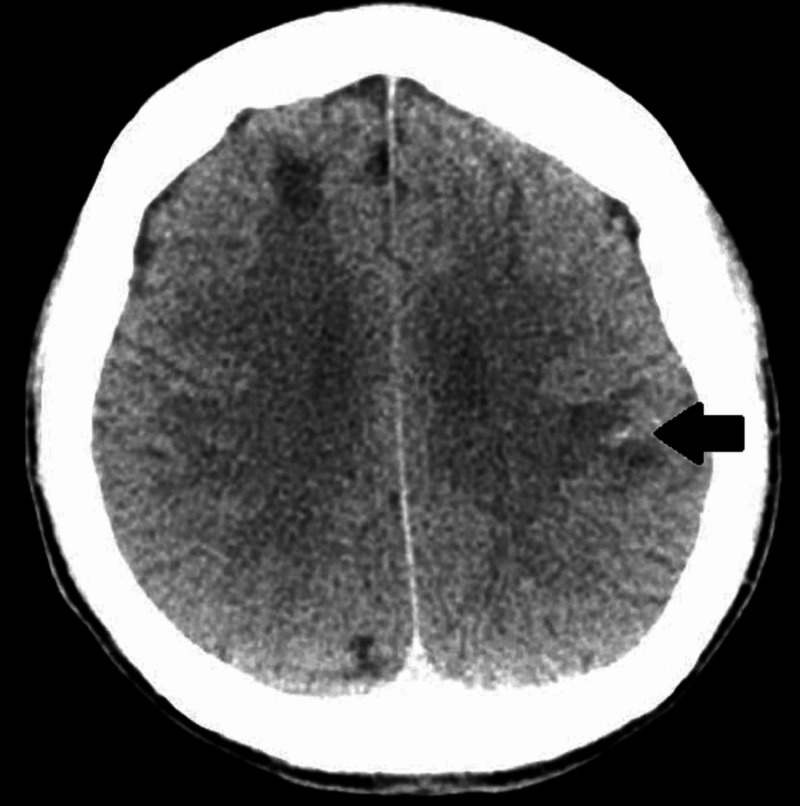
Computed tomography of head showing punctate hypointensity in the left temporoparietal region (black arrow)

Complete blood count was significant for an elevated white blood cell count of 17.78 k/uL (normal: 3.7-10.3 k/uL). CD4 cell count was less than 10/uL, while he was not on ART (normal: 490-1730/uL). Computed tomography (CT) scan of the head showed multiple non-specific hypoattenuating subcortical white matter lesions in bilateral cerebral hemispheres, ventriculomegaly, old post-operative changes in the posterior fossa, and punctate hyperdensity in the left temporoparietal region (Figure 1).

**Figure 2:**
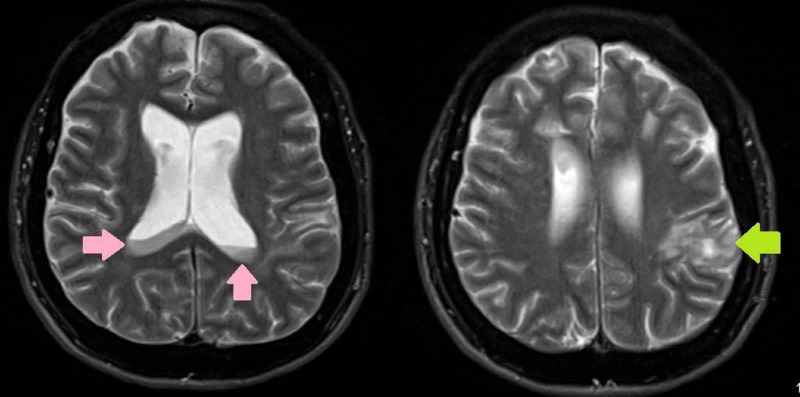
Magnetic resonance imaging of the brain showing pus in the ventricles (pink arrows) and a developing abscess in the left parietal lobe (green arrow)

Electroencephalographic (EEG) testing was normal on presentation. Blood cultures grew Salmonella and methicillin-sensitive Staphylococcus aureus (MSSA), and the patient was started on intravenous ceftriaxone. MSSA was thought of as a skin contaminant, hence not treated. Magnetic resonance imaging (MRI) of the brain was concerning for pus in ventricles with re-demonstration of multiple acute infarcts in bilateral frontal lobes and deep nuclei and a developing abscess in the left parietal lobe (Figure 2).

CT scan of the abdomen and pelvis showed no intra-abdominal source of infection. An echocardiogram showed no vegetation, and a lumbar puncture was performed.

Relevant differential diagnoses include acute encephalopathy due to sepsis secondary to bacteremia or central nervous system (CNS) infections such as toxoplasmosis, Cryptococcus, CNS lymphoma, or ischemic stroke.

The patient was commenced on intravenous ceftriaxone 2 grams every 12 hours for Salmonella coverage. Trimethoprim-sulfamethoxazole was started for Pneumocystis prophylaxis and azithromycin for Mycobacterium avium complex prophylaxis. The patient’s HIV ribonucleic acid (RNA) was elevated to 150251 copies/mL, wild genotype. Cryptococcal antigen testing was negative, and Toxoplasma IgG was 5.14 IU/mL (9 IU/mL or less is considered negative). ART was not started due to concerns about immune reconstitution inflammatory syndrome. The cerebrospinal fluid (CSF) analysis was suggestive of bacterial meningitis, and the CSF culture grew Salmonella spp. (Table 1).

**Table 1:** Cerebrospinal fluid analysis after lumbar puncture nl: normal level

Cerebrospinal fluid analysis	
Appearance	Cloudy
Nucleated cells	>10000 (nl: 0-5/uL)
Neutrophils	>9400/uL (94%)
Glucose	1 mg/dL (nl:41-70 mg/dL)
Total protein	315 mg/dL (nl: 15-45 mg/dL)
Cryptococcal antigen	Negative
Acid-fast stain	Negative
Culture	Salmonella spp.

**Figure 3:**
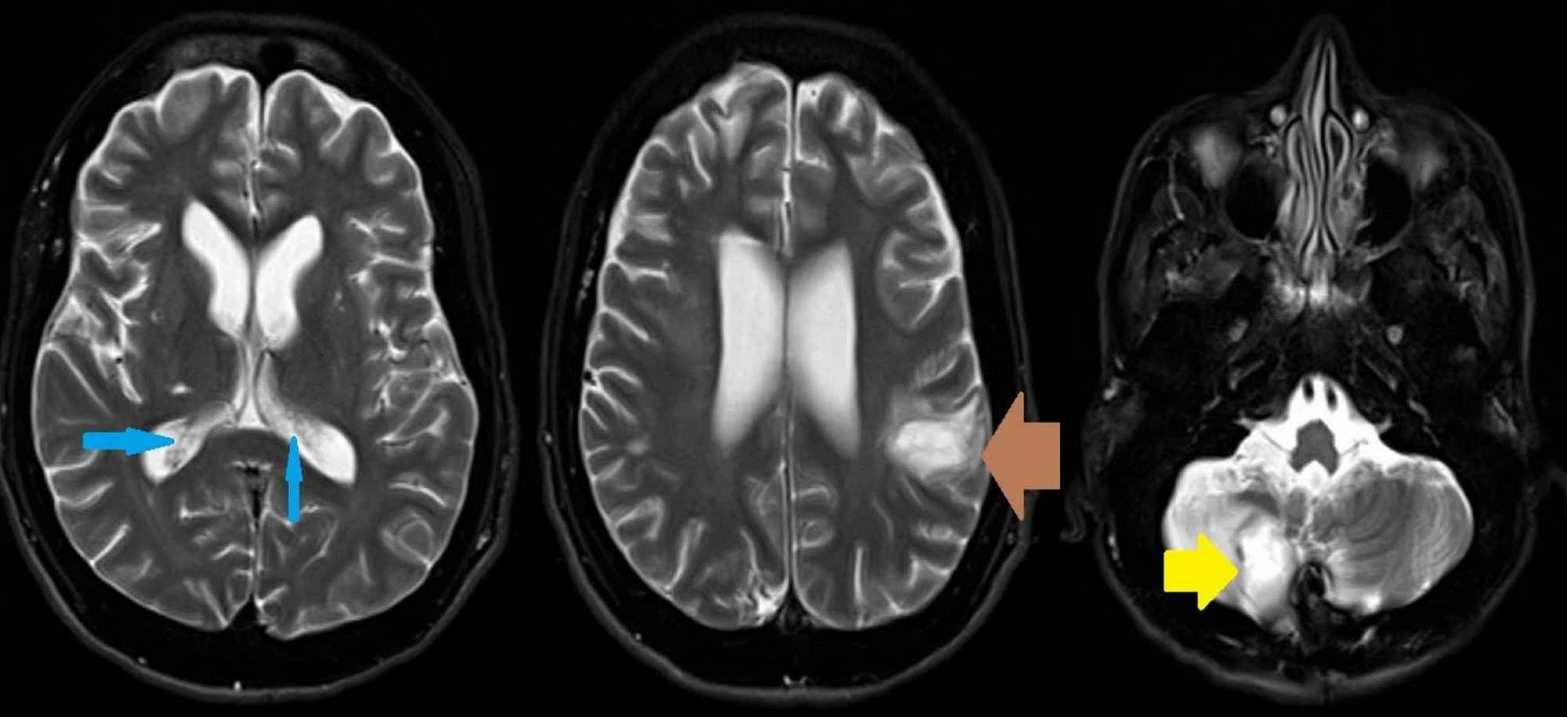
Magnetic resonance imaging of the brain showing ventriculitis (blue arrows), enlarged left parietal abscess (brown arrow), and right cerebellar abscess (yellow arrow)

Syphilis immunoglobulin testing was negative, as was the hepatitis panel. QuantiFERON-TB Gold Plus (Qiagen, Hilden, Germany) testing showed an intermediate result. Repeat lumbar puncture was performed to assess opening pressures. The patient continued to spike fevers of 103.2°F and got progressively obtunded. Repeat CT head with contrast revealed worsening ventriculitis, enlarged left parietal abscess (3.2 x 1.8 cm), stable right cerebellar abscess (2.8 x 2.3 cm), and cerebral edema (Figure 3).

The patient underwent right suboccipital mini-craniectomy and left frontoparietal craniectomy with ultrasound-guided intra-operative neuronavigation for the removal of abscess. Cerebellar abscess culture also grew Salmonella species. The repeat blood and CSF blood cultures cleared on antibiotic therapy. The fevers resolved after the surgical drainage of abscesses, and the patient eventually had a peripherally inserted central catheter placed. The patient's aphasia continued to improve during the hospital stay with rehabilitation. The patient was discharged on outpatient antibiotic therapy with intravenous ceftriaxone for a total of six weeks duration.

## Discussion

Salmonella infection is usually known to be associated with foodborne diarrhea worldwide, and the incidence of Salmonella meningitis is rare, only 4-6 per 100,000/year, especially in the immunocompromised hosts [5]. Although any of the pyogenic bacteria may cause brain abscess, brain abscesses caused by Salmonella species are extremely rare [6]. Numerous predisposing conditions such as prior neurologic procedures, primary or metastatic brain tumors, neurologic procedures, steroid use, diabetes mellitus, and HIV infection have been described. 

A defective cell-mediated immunity is postulated to pre-dispose HIV infected patients to invasive Salmonella infections. An intact regulatory cytokine cascade is important to defend against Salmonella, as demonstrated by the observation that patients with isolated interleukin-12 receptor defects are at increased risk of particularly severe Salmonella infections [1]. 

The location of abscesses caused by Salmonella and the associated clinical features described in the literature are similar to those with pyogenic brain abscesses due to any other organism. Early imaging of the nervous system, as well as diagnostic aspiration, are required to make a prompt diagnosis [7]. Our case was unique in the aspect that Salmonella meningitis was associated with brain abscess as well as ventriculitis in an adult patient.

Ventriculitis is defined as a suppurative infection of the ventricles of the brain, usually occurring secondary to shunt or catheter-related infections. Ventriculitis secondary to Salmonella species is not reported in medical literature to the author's knowledge. Magnetic resonance imaging features of ventriculitis include ventricular debris, hydrocephalus, periventricular hyperintense signals, and ependymal enhancement [8], as evident in our case (Figure 2).

The early administration of antimicrobial therapy combined with surgical drainage is associated with the best probability of cure [9]. Owing to their excellent central nervous system penetration, third generation cephalosporins are the most consistently used agents. Chloramphenicol is postulated to be considered as an alternative, with good CNS penetration and activity against Salmonella but is not available in the United States. Mortality rates are high (approaching 40-60%), especially when Salmonella meningitis is associated with brain abscess, and permanent neurologic damage is reported in a significant number of survivors. Prognosis is most favorable when the patients receive prompt antimicrobial therapy and surgical treatment [8]. 

## Conclusions

Infection with Salmonella species is a rare and infrequent cause of meningitis. It is commonly reported in the pediatric population but can also affect adults with immunocompromised conditions such as HIV infection. Our patient appears to be among the first reported cases of ventriculitis caused by Salmonella in an adult patient. Early diagnosis and management with surgical drainage along with antibiotic therapy seem to improve prognosis in this highly fatal condition. 
